# Facility-Level Factors Influencing Retention of Patients in HIV Care in East Africa

**DOI:** 10.1371/journal.pone.0159994

**Published:** 2016-08-10

**Authors:** Beth Rachlis, Giorgos Bakoyannis, Philippa Easterbrook, Becky Genberg, Ronald Scott Braithwaite, Craig R. Cohen, Elizabeth A. Bukusi, Andrew Kambugu, Mwebesa Bosco Bwana, Geoffrey R. Somi, Elvin H. Geng, Beverly Musick, Constantin T. Yiannoutsos, Kara Wools-Kaloustian, Paula Braitstein

**Affiliations:** 1 The Ontario HIV Treatment Network, Toronto, Canada; 2 Division of Clinical Public Health, Dalla Lana School of Public Health, University of Toronto, Toronto, Canada; 3 Department of Biostatistics, Richard Fairbanks School of Public Health, Indiana University, Indianapolis, Indiana, United States of America; 4 Infectious Diseases Institute, Kampala, Uganda; 5 Department of Health Services, Brown University, Providence, Rhode Island, United States of America; 6 Department of Population Health, School of Medicine, New York University, New York, New York, United States of America; 7 Department of Obstetrics, Gynecology & Reproductive Sciences, University of California San Francisco, San Francisco, California, United States of America; 8 Centre for Microbiology Research, Kenya Medical Research Institute, Nairobi, Kenya; 9 Department of Internal Medicine, Mbarara University of Science & Technology, Mbarara, Uganda; 10 National AIDS Control Programme, Dar es Salaam, Tanzania; 11 Department of Medicine, University of California San Francisco, San Francisco, California, United States of America; 12 Department of Biostatistics, Indiana University School of Medicine, Indianapolis, Indiana, United States of America; 13 Division of Infectious Diseases, Department of Medicine, Indiana University School of Medicine, Indianapolis, Indiana, United States of America; 14 Academic Model Providing Access To Healthcare (AMPATH), Eldoret, Kenya; 15 Department of Medicine, School of Medicine, College of Health Sciences, Moi University, Eldoret, Kenya; 16 Division of Epidemiology, Dalla Lana School of Public Health, University of Toronto, Toronto, Canada; Katholieke Universiteit Leuven Rega Institute for Medical Research, BELGIUM

## Abstract

Losses to follow-up (LTFU) remain an important programmatic challenge. While numerous patient-level factors have been associated with LTFU, less is known about facility-level factors. Data from the East African International epidemiologic Databases to Evaluate AIDS (EA-IeDEA) Consortium was used to identify facility-level factors associated with LTFU in Kenya, Tanzania and Uganda. Patients were defined as LTFU if they had no visit within 12 months of the study endpoint for pre-ART patients or 6 months for patients on ART. Adjusting for patient factors, shared frailty proportional hazard models were used to identify the facility-level factors associated with LTFU for the pre- and post-ART periods. Data from 77,362 patients and 29 facilities were analyzed. Median age at enrolment was 36.0 years (Interquartile Range: 30.1, 43.1), 63.9% were women and 58.3% initiated ART. Rates (95% Confidence Interval) of LTFU were 25.1 (24.7–25.6) and 16.7 (16.3–17.2) per 100 person-years in the pre-ART and post-ART periods, respectively. Facility-level factors associated with increased LTFU included secondary-level care, HIV RNA PCR turnaround time >14 days, and no onsite availability of CD4 testing. Increased LTFU was also observed when no nutritional supplements were provided (pre-ART only), when TB patients were treated within the HIV program (pre-ART only), and when the facility was open ≤4 mornings per week (ART only). Our findings suggest that facility-based strategies such as point of care laboratory testing and separate clinic spaces for TB patients may improve retention.

## Introduction

The number of individuals accessing treatment for HIV has increased markedly over the last decade, particularly in sub-Saharan Africa (SSA) [1,2]. This has resulted in significant decreases in morbidity and in mortality of around 32% between 2005 and 2014 among people living with HIV (PLHIV) in SSA [1, 2]. However, engagement along all stages of the HIV cascade of care is needed in order to achieve and maintain viral suppression and prevent new infections [3,4]. Disruptions in care may undermine any individual gains in clinical outcomes [5] and the interruption of ART can lead to treatment failure, and associated drug resistance with disease progression [6]. Indeed, studies of patients traced following loss to follow-up (LTFU) have reported higher rates of mortality compared to those retained in care [7]. Identifying the various patient and structural factors that can increase risk of loss to follow-up are needed to inform strategies that promote retention in care.

Recently, the Joint United Nations Programme on HIV/AIDS (UNAIDS) have endorsed new global fast-track targets: 90% of all PLHIV to be diagnosed and know their status, 90% of all people diagnosed with HIV infection to receive ART, and 90% of those receiving ART to be virally suppressed by 2020 [8]. Retention in care from enrolment through ART-initiation and beyond is critical for achieving these targets. Unfortunately, LTFU in both the pre- and post-ART periods remains a key challenge for HIV programs. It is estimated that in sub-Saharan Africa, less than half of individuals are retained in pre-ART care [9] and median retention at 3 years on ART has been shown to be 65–70% [10].

Various patient factors have been associated with becoming LTFU which include demographic (e.g., gender, age) [11–13] and clinical or laboratory characteristics (e.g., stage of disease or CD4 count) [14] as well as socio-economic (e.g., transport costs and income) [13–15] and social factors (e.g., marital status) [13, 15]. While some attention has been paid to exploring the facility-level factors that can affect LTFU rates including where care is located (e.g., decentralized care), the type and model of care (e.g., hospital-based versus primary health centres), staffing characteristics, and the role of patient-provider relationships [5, 11, 12, 15], further study is needed to identify strategies and interventions that could be adopted at the facility or program level to improve retention in all stages of the HIV care cascade [5]. Using East African International epidemiologic Databases to Evaluate AIDS (EA-IeDEA) data, the objective of the present study was to explore facility-level factors that are associated with LTFU in the pre- and post-ART periods among patients receiving HIV care in Kenya, Uganda and Tanzania.

## Materials and Methods

### Study population: East African International epidemiologic Databases to Evaluate AIDS (EA-IeDEA)

This retrospective cohort study involved patient-level and facility-level data from 29 sites affiliated with the East Africa (EA)-IeDEA Consortium. EA-IeDEA is a cohort of patients from HIV clinical care sites in Uganda, Kenya and Tanzania. Data are collected in the context of routine care at baseline and each follow-up visit, including socio-demographic data, the date of starting ART, type of treatment initiated, and, where available, CD4 counts and HIV-1 plasma RNA levels at baseline and during follow-up. All programs collect pre-ART data. Many programs may also collect contact information to facilitate patient tracing although not all programs trace patients who become LTFU. Tracing methods for missed visits vary between sites and may include mobile phone calls, SMS reminders, and/or home visits. Some clinics also involve volunteers from community-based organizations (CBO) to track patients.

This analysis was limited to all adult patients, 14 years of age or older who were enrolled in a clinic associated with EA-IeDEA between May 2000 and May 2009. The exact date of database closure differed by program and ranged from 31 March 2008 to 19 May 2009. We excluded pregnant women from our analyses, mainly because of the variability in the frequency of follow-up for pregnant women and their movement between antenatal care (ANC) and HIV care which can affect retention. Indeed, previous studies have demonstrated higher rates of LTFU among pregnant women compared to non-pregnant women and other populations [16–18].

In Kenya, participating programs included the United States Agency for International Development-Academic Model Providing Access to Healthcare (USAID-AMPATH), the Family AIDS Care and Education Services (FACES), and the MTCT (Mother-to-Child Transmission)-Plus program in Kisumu. In Uganda, affiliated sites included the Infectious Diseases Institute (IDI), Mbarara Immune Suppression Syndrome (ISS) clinic and the Makerere University-Johns Hopkins University (MU-JHU) collaboration at Mulago Hospital (MTCT-Plus program). Contributing sites in Tanzania included the Tumbi Regional Hospital in Kibaha, the Ocean Road Cancer Institute in Dar es Salaam, and the Morogoro Regional Hospital in Morogoro. Facility data came from a survey of all participating facilities conducted between August 2009 and February 2010 [19]. All analysis was performed with de-identified data and presented at the country or program level. The IeDEA protocol was approved by the Indiana University IRB and all local regulatory bodies involved in this consortium. Patient level consent was waived by all regulatory entities because the data were de-identified prior to analysis and all data were routinely collected as part of patient care.

### Measures

A socio-ecological framework was developed to guide our hypotheses about what measures to include in this analysis [20]. In this framework, the probability of becoming LTFU is a combination of individual patient factors (e.g., demographic, socio-economic and clinical characteristics), healthcare facility factors (e.g., location, operations and strategies used for retention) and broader contextual factors including the national context (e.g., policies, infrastructure, environment). Note, only patient-level and facility-level factors were available and explored in the present study.

Loss to follow-up, the primary outcome, was defined as no clinic visits for at least 12 months for the pre-ART period and at least 6 months for the post-ART period, immediately prior to database closure. Six months has previously been proposed as the most informative definition of LTFU for individuals on ART [21]. For individuals not yet on ART, routine follow-up may be less frequent (e.g., 3 to 6 months) suggesting that such individuals would be considered LTFU if they missed two to four visits.

Patient-level measures included demographic factors: age at enrolment (in years) and gender (male/female); socio-economic characteristics: education level (none, some primary, some secondary, some college/university/technical), disclosure of HIV status (yes/no), and travel time to clinic (<30 minutes, 30–60 minutes, 1–2 hours, >2 hours); and clinical characteristics: on ART (yes/no), time on ART (in months), CD4 count at enrolment (cells/μl), CD4 count at ART initiation (<201, 201–350, 351–500, 501–650, >650), World Health Organization (WHO) clinical stage at enrolment (pre-ART analysis) and/or at ART initiation (ART analysis) (Stage 1–4), ever on TB treatment (yes/no) and calendar year at enrollment or ART initiation (2000–2006; 2007–2009). Two periods were selected to account for the rapid expansion of highly active antiretroviral therapy in East Africa.

Since little is known about how facility factors can influence retention in HIV care, we explored a range of available facility-level characteristics. We used our conceptual model to categorize facility factors of interest. General facility characteristics included location (urban, rural, semi-urban), HIV lab availability including HIV RNA PCR (on site vs. off site vs. not available) and CD4 count (on site vs. off site) as well as the turnaround time for HIV RNA PCR (≤14 days vs. >14 days) and CD4 count (≤7 days vs. >7 days). Availability of non- HIV labs (on site vs. off site) which included total lymphocytes, ALT/AST, and creatinine were also recorded as well as disruptions in CD4 reagents in the last year (no vs. yes). The level of care including whether the facility was considered primary (e.g., community health centres, dispensaries), secondary (e.g., district hospitals) or tertiary (e.g., regional hospitals, referral hospitals) was also explored. Clinical operations included operating hours: open ≤4 mornings vs. >4 mornings during the week, open ≤ 4 evenings vs. >4 evenings during the week, and open on weekends (no vs. yes); presence of an electronic database to capture data on patients who transfer in (no vs. yes) and transfer out (no vs. yes); a waitlist for initiating ART (no vs. yes); daily average number of available physicians/mid-level staff (<3 vs. ≥3); ratio of adult patients to daily number of physicians/mid-level staff (<10 vs. ≥10); daily average number of lay outreach workers (<2 vs. ≥2); the ratio of adult patients to number of lay outreach workers (<10 vs. ≥10); whether cotrimoxazole prophylaxis, food rations/supplements, vitamins (including zinc), and nutritional treatment is provided (no vs. yes) and whether TB is diagnosed and treated within the ART program (yes vs. symptomatic patients referred to TB clinic). Retention strategies included whether pre-ART outreach services were available (no vs. yes), the number of days to track patients after a missed visit (≤7 days vs. >7 days), whether outreach workers phone missing patients first (no vs. yes), if a business number (no vs. yes) or a relative’s number (no vs. yes) was available, availability of home visits (no vs. yes), type of transport for home visits (all available vs. bike/foot/public vs. motor vehicle only) and death ascertainment through active outreach (no vs. yes). While broader national contextual factors also influence LTFU, we were unable to measure such factors directly.

### Statistical analysis

Categorical variables were described using frequencies and proportions. Description of the quantitative variables was based on the median and interquartile range (IQR). Rates of LTFU were estimated using the maximum likelihood under an exponential distribution assumption, and the associated standard errors were calculated based on non-parametric bootstrap methodology. Modelling the cause-specific hazard of LTFU was based on the semi-parametric proportional hazards model, with a gamma-distributed shared frailty according to EA-IeDEA program to take into account the association between patients from the same EA-IeDEA program. Pre-ART and post-ART periods were analysed separately (S1 File). Note that an interaction term for use of vitamin and nutritional supplements was also explored. The time origin for the pre-ART analysis was enrolment date whereas ART initiation date was used for the post-ART analysis. Follow-up was right-censored at ART initiation or database closure date (whichever came first) for the pre-ART analysis and database closure date for the post-ART analysis. Death (for both analyses) and ART initiation (for pre-ART analysis) were considered as competing risks to LTFU. Variable selection was performed in two phases: First patient-level variables were selected using a backwards elimination procedure (while keeping in the model age and gender regardless of the significance level) and second, conditional on the selected patient-level variables, the facility-level variables were selected again using backwards variable elimination procedure.

The date of death was missing for 718 (13.7%) of the 5,256 deaths that occurred in this sample. In order to deal with missing death times, we performed hot-deck imputation [22] under the assumption of missing at random (MAR). In this method, each patient with a missing death date is associated with a set of similar patients with known death dates (donor pool). The key advantage of this method is that it is non-parametric, which means that it does not make any distributional assumptions about the missing data. To make the MAR assumption plausible, we evaluated factors affecting both the probability of missingness in the date of death and also the death time. The covariates used to form the donor pools for imputing the missing death times (recipient pool) were age category at last clinic visit, gender, CD4 cell count at enrolment, WHO stage at enrolment and the indicator of being on ART at the last clinic visit. In cases with missing CD4 or WHO stage, those variables were not used in creating donor pools. Standard error (SE) estimation was based on non-parametric bootstrap SE estimator (20 replications), taking into account the additional source of uncertainty due to imputing the missing death times [22]. Since the sample size was large, standard asymptotic distribution results were used for 95% confidence intervals (CI) and p-value calculations. Statistical analysis was performed using the Stata 13 for Windows statistical package.

## Results

In total, the analysis included 88,152 patients enrolled in one of the 29 clinics from 9 programs within the EA-IeDEA Consortium. The majority were female (68.3%) and 10,790 (18.0%) women were pregnant at some point during the follow-up period. After excluding pregnant women, the final sample size consisted of 77,362 patients.

### Patient and facility-level characteristics

Demographic and clinical characteristics of the study sample are presented in Table 1. Briefly, the overall median (IQR) age at enrolment was 36.0 (30.1, 43.1). More than half of the patients (58.3%) initiated ART at some point during their follow-up. Median CD4 levels around ART initiation were similar for the three countries (median range: 105–109 cells/μl).

**Table 1:** Patient characteristics of included patients at all EA-IeDEA sites.

	East Africa IeDEA Country			
	Kenya	Tanzania	Uganda	Overall
	N (%)	N (%)	N (%)	N (%)
**Gender**				
*Female*	38178 (63.9)	5402 (67.7)	5733 (59.5)	49313 (63.7)
*Male*	21354 (35.7)	2575 (32.3)	3910 (40.5)	27839 (36.0)
*Missing*	210 (0.4)	0 (0.0)	0 (0.0)	210 (0.3)
**HIV status disclosed**				
*No*	18646 (31.2)	0 (0.0)	153 (1.6)	18799 (24.3)
*Yes*	33296 (55.7)	0 (0.0)	616 (6.4)	33912 (43.8)
*Missing*	7800 (13.1)	7977 (100.0)	8874 (92.0)	24651 (31.9)
**Highest education level achieved**				
*None*	1388 (2.3)	0 (0.0)	205 (2.1)	1593 (2.1)
*Some primary*	29954 (50.1)	0 (0.0)	829 (8.6)	30783 (39.8)
*Some secondary*	15997 (26.8)	0 (0.0)	418 (4.3)	16415 (21.2)
*Some College/University or technical*	3164 (5.3)	0 (0.0)	178 (1.8)	3342 (4.3)
*Missing*	9239 (15.5)	7977 (100.0)	8013 (83.1)	25229 (32.6)
**Travel time to clinic**				
*< 30 minutes*	14566 (24.4)	0 (0)	205 (2.1)	14771 (19.1)
*30–60 minutes*	16983 (28.4)	0 (0)	459 (4.8)	17442 (22.5)
*1–2 hours*	12918 (21.6)	0 (0)	258 (2.7)	13176 (17.0)
*> 2 hours*	9165 (15.3)	0 (0)	186 (1.9)	9351 (12.1)
*Missing*	6110 (10.2)	7977 (100.0)	8535 (88.5)	22622 (29.2)
**Ever on ART**	31361 (52.5)	4281 (53.7)	9441 (97.9)	45083 (58.3)
**CD4 at ART start (90 days pre—7 post) cells/**μl				
*<201*	19040 (60.7)	2537 (59.2)	4586 (48.6)	26163 (58.0)
*201–350*	3213 (10.2)	427 (10.0)	697 (7.4)	4337 (9.6)
*351–500*	770 (2.5)	103 (2.4)	81 (0.9)	954 (2.1)
*501–650*	345 (1.1)	32 (0.7)	45 (0.5)	422 (0.9)
*>650*	329 (1.0)	15 (0.3)	29 (0.3)	373 (0.8)
*Missing*	7664 (24.4)	1175 (27.4)	4003 (42.4)	12842 (28.5)
**WHO stage at ART start (90 days pre—7 post)**				
*1*	4897 (18.8)	121 (4.8)	307 (3.7)	5325 (14.4)
*2*	5395 (20.7)	664 (26.3)	2374 (28.5)	8433 (22.8)
*3*	13046 (50.0)	980 (38.8)	3408 (40.9)	17434 (47.2)
*4*	2747 (10.5)	764 (30.2)	2238 (26.9)	5749 (15.6)
**TB treatment (at any time)**	13218 (22.1)	556 (7.0)	3059 (31.7)	16833 (21.8)
	**Median (IQR)**	**Median (IQR)**	**Median (IQR)**	**Median (IQR)**
**Age at enrollment (years)**	35.9 (29.9, 43.2)	36.6 (30.6, 44.0)	36.0 (30.7, 41.7)	36.0 (30.1, 43.1)
**CD4 at enrollment (w/in 3 mos) cells/**μl	198 (80, 381)	151 (60, 306)	131 (45, 245)	185 (74, 359)
**CD4 at ART start (90 days pre—7 post) cells/**μl	109 (45, 183)	105 (39, 178)	107 (36, 176)	109 (43, 181)
	Median* **(95% CI)**	Median* **(95% CI)**	Median* **(95% CI)**	Median* **(95% CI)**
**Pre-ART follow up duration (years)**	0.34 (0.33, 0.35)	0.20 (0.19. 0.21)	0.32 (0.31, 0.33)	0.32 (0.31, 0.33)
**ART follow up duration (years)**	1.57 (1.56. 1.59)	1.26 (1.20, 1.32)	2.68 (2.53, 2.82)	1.66 (1.64, 1.68)

* Calculated using the Kaplan-Meier estimator by considering right censoring as the event of interest.

A description of the key facility-level characteristics is given in Table 2. Only a small fraction of the clinics were located in rural areas (13.8%) and a minority provided tertiary level of care (20.7%). While more than half of the clinics were open more than four mornings and more than four evenings during the week, the majority (96.6%) were not open on the weekends. Most of the clinics did not have a wait list for initiating ART (75.9%). The majority of clinics provided nutritional supplements (79.3%) and food rations (75.9%). Only a minority of facilities had on-site HIV RNA PCR (17.2%) and CD4 count (31.0%) testing, and turnaround time for HIV RNA PCR results was >14 days for the majority of clinics. The vast majority of sites had at least two outreach workers (82.8%) and outreach efforts were initiated within a week after a missed clinic visit in most cases (79.3%). The ratio of adult patients to the number of outreach workers was at least 10 to 1 in 75.9% of clinics. Death was ascertained through active outreach methods in the majority of sites (82.8%).

**Table 2:** Facility-level characteristics of included facilities (n = 29).

GENERAL FACILITY CHARACTERISTICS	n (%)
**Location**	
***Urban***	12 (41.4)
***Rural***	4 (13.8)
***Semi-urban***	13 (44.8)
**Level of care**	
***Primary***	10 (34.5)
***Secondary***	13 (44.8)
***Tertiary***	6 (20.7)
**HIV RNA PCR: Availability**	
***On Site***	5 (17.2)
***Off Site***	22 (75.9)
***Test Not Available***	2 (6.9)
**HIV RNA PCR turnaround time**	
***< = 14 days***	9 (31.0)
***>14 days***	17 (58.6)
***Unknown/missing***	3 (10.3)
**CD4 count: Availability**	
***On Site***	9 (31.0)
***Off Site***	20 (69.0)
**CD4 turnaround time**	
***< = 7 days***	14 (48.3)
***>7 days***	15 (51.7)
**Disruption in CD4 reagents in last year**	
***Yes***	8 (27.6)
***No***	21 (72.4)
Non-HIV labs*	
***On Site***	10 (34.5)
***Off Site***	19 (65.5)
**CLINICAL OPERATIONS**	
**Open in mornings**	
***< = 4 mornings***	11 (37.9)
***>4 mornings***	18 (62.1)
**Open in evenings**	
***< = 4 evenings***	13 (44.8)
***>4 evenings***	16 (55.2)
**Open on weekends**	
***No***	28 (96.6)
***Yes***	1 (3.4)
**Waiting list for initiating ART**	
***Yes***	7 (24.1)
***No***	22 (75.9)
**Electronic Database to capture patients transferred in**	
***No***	20 (69.0)
***Yes***	9 (31.0)
**Electronic database to capture patients transferred out**	
***Yes***	24 (82.8)
***Not applicable***	5 (17.2)
**Daily average number of physicians/mid-level staff**	
***<3***	11 (37.9)
** ≥ 3**	18 (62.1)
**Ratio of adult patients to physicians/mid-level staff**	
***<10***	8 (27.6)
**≥*10***	21 (72.4)
**Daily average number of lay outreach workers**	
***<2***	5 (17.2)
**≥*2***	24 (82.8)
**Ratio of adult patients to lay outreach workers**	
***<10***	7 (24.1)
**≥*10***	22 (75.9)
**Vitamins (including ZINC) provided**	
***No***	20 (69.0)
***Yes***	9 (31.0)
**Nutritional supplements**	
***No***	6 (20.7)
***Yes***	23 (79.3)
**Food rations**	
***No***	7 (24.1)
***Yes***	22 (75.9)
**Cotrimoxazole prophylaxis prescribed**	
***No***	22 (75.9)
***Yes***	7 (24.1)
**TB diagnosed and treated within ART program**	
***Yes*, *at ART initiation and regularly thereafter***	19 (65.5)
***No*, *symptomatic patients are referred to the TB clinic***	10 (34.5)
**RETENTION STRATEGIES**	
**Pre-ART outreach program**	
***No***	15 (51.7)
***Yes***	14 (48.3)
**days to track patient after missed visit**	
***>7***	6 (20.7)
***< = 7***	23 (79.3)
**Phone first for outreach**	
***No***	3 (10.3)
***Yes***	26 (89.7)
**Business number available**	
***No***	12 (41.4)
***Yes***	17 (58.6)
**Relative number available**	
***No***	8 (27.6)
***Yes***	21 (72.4)
**Home visits available**	
***No***	2 (6.9)
***Yes***	27 (93.1)
**Type of transport for home visits**	
***All available***	22 (78.6)
***Bike/Foot/Pub***	3 (10.7)
***Car only***	2 (7.1)
***N/A***	1 (3.6)
**Death ascertained through active outreach**	
***No***	5 (17.2)
***Yes***	24 (82.8)

* Non-HIV labs include total lymphocytes, total ALT/AST and creatinine.

### Frequency and predictors of LTFU

**Fig 1:**
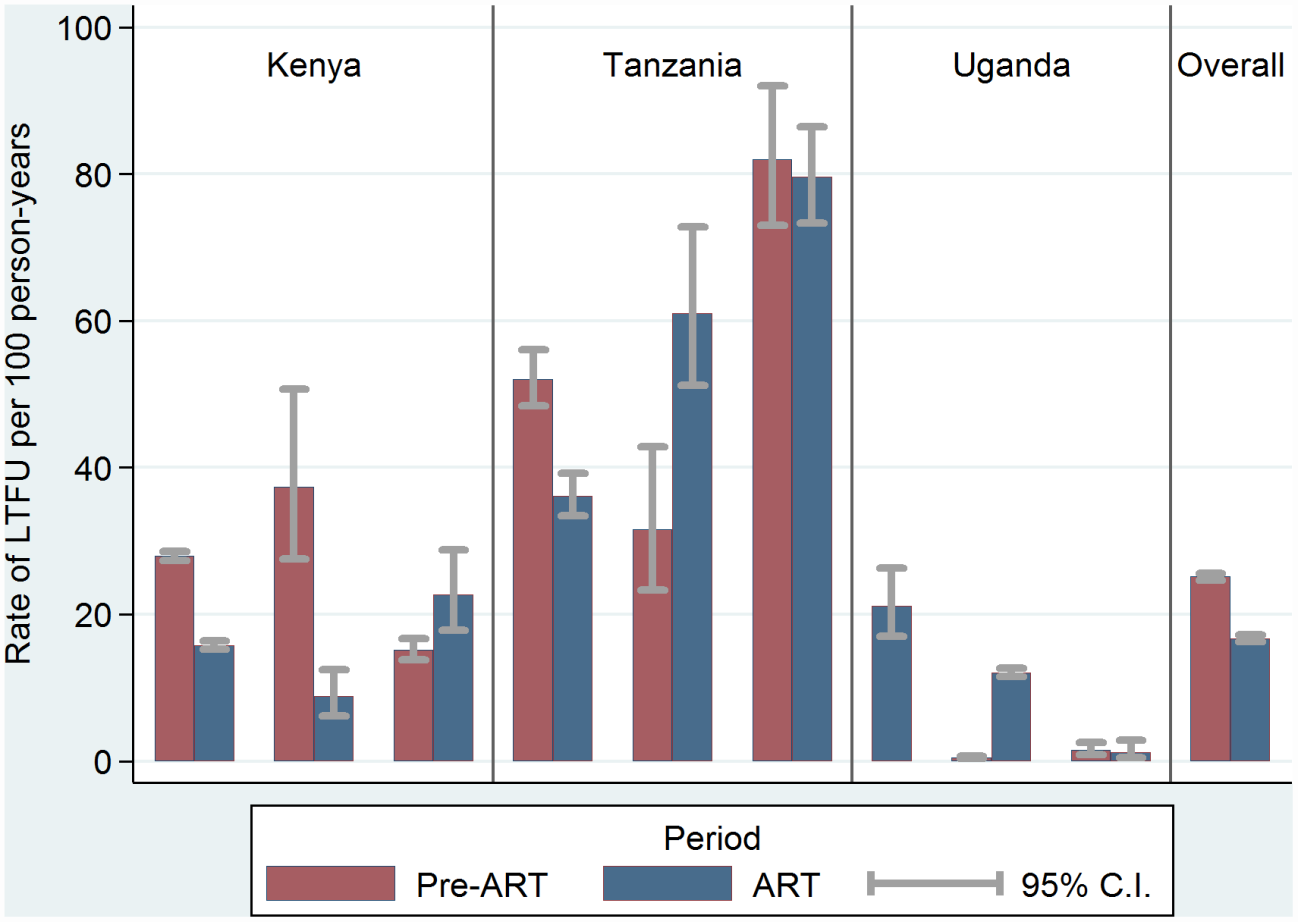
Rates of loss to follow up (LTFU) by country and EA-IeDEA program.

Pre-ART and post-ART rates of LTFU by EA-IeDEA program are presented in Fig 1. The overall rates (95% Confidence Intervals (CI)) of LTFU were 25.1 (24.7–25.6) and 16.7 (16.3–17.2) per 100 person-years in the pre-ART and post-ART analysis, respectively. Programs from Tanzania reported the highest rates of LTFU (95% CI): 62.3 (57.9–67.2) and 53.2 (50.4–56.0) for the pre-ART and post-ART analysis, respectively. LTFU was lowest in Ugandan programs particularly in the pre-ART period: 0.6 (0.5–0.7) and 11.9 (11.4–12.6). The corresponding figures for Kenya were 27.0 (26.4–27.7) per 100 person-years in the pre-ART period and 15.9 (15.3–16.4) in the ART period. Additionally, significant variability was observed regarding rates of LTFU among EA-IeDEA programs within the same country.

Results from the multivariable analysis of LTFU both pre- and post-ART initiation are presented in Table 3. Secondary-level care was associated with a decreased hazard of LTFU both pre-ART and post-ART (adjusted hazard ratios- AHRs (95% CI): 0.81 (0.75–0.88) and 0.76 (0.71–0.81), respectively). Nutritional supplements (0.62, 0.44–0.87) and referring TB patients to a separate TB clinic (versus treating them within the ART program) (0.91, 0.83–0.99) both had favourable impacts on LTFU in the pre-ART period only. Rates of LTFU were higher in facilities without CD4 tests available on-site and with longer HIV-RNA PCR turnaround times, in both analysis periods (pre-ART 1.21 (1.11–1.33) and 1.14 (1.07–1.20), respectively; post-ART: 1.19 (1.05–1.34) and 1.29 (1.19–1.40), respectively). Being open more than 4 mornings per week was associated with a decreased hazard of LTFU (0.75, 0.62–0.90) in the ART period.

**Table 3:** Factors associated with cause-specific hazard of loss to follow-up from enrollment (Pre-ART) and after ART initiation in multivariate analysis.**

	*Pre-ART* (n = 58,727)			*ART* (n = 35,041)		
	HR	95% CI	p-value	HR	95% CI	p-value
**Level of care**						
*Primary*	1			1		
*Secondary*	0.81	(0.75, 0.88)	<0.001	0.76	(0.71, 0.81)	<0.001
*Tertiary*	0.938	(0.79, 1.09)	0.36	1.32	(1.10, 1.58)	0.002
**Nutritional supplements**						
*No*	1			1		
*Yes*	0.62	(0.44, 0.87)	0.006	1.22	(0.71, 2.09)	0.480
**HIV RNA PCR turnaround time**						
*< = 14 days*	1			1		
*>14 days*	1.14	(1.07, 1.20)	<0.001	1.29	(1.19, 1.40)	<0.001
*Unknown/missing*	1.47	(1.10, 1.95)	0.008	2.64	(2.01, 3.45)	<0.001
**CD4 count: Availability**						
*On Site*	1			1		
*Off Site*	1.21	(1.11, 1.33)	<0.001	1.19	(1.05, 1.34)	0.005
**Identify pts transferred in (electronic database)**						
*No*	1					
*Yes*	1.36	(0.88, 2.12)	0.171	-	-	-
**Have waiting list for initiating ART for adult patients followed at this facility**						
*Yes*	1			1		
*No*	0.99	(0.70, 1.41)	0.97	0.90	(0.57, 1.40)	0.631
**TB diagnosed and treated by clinicians within the ART program**						
*Yes*, *at ART initiation and regularly thereafter*	1			1		
*No*, *symptomatic patients are referred to the TB clinic*	0.91	(0.83, 0.99)	0.044	0.91	(0.79, 1.05)	0.181
**Open in mornings**						
*< = 4 mornings*				1		
*>4 mornings*	-	-	-	0.75	(0.62, 0.90)	0.003
**Open in evenings**						
*< = 4 evenings*	1			1		
*>4 evenings*	0.92	(0.85, 1.00)	0.054	1.21	(0.98, 1.49)	0.083
**Phone first for outreach**						
*No*				1		
*Yes*	-	-	-	0.85	(0.59, 1.22)	0.377
**Male gender**						
*No*	1			1		
*Yes*	1.07	(1.03, 1.12)	0.002	1.04	(0.98, 1.09)	0.164
**Age at enrollment**						
*<25*	1			1		
*25–34*.*9*	0.68	(0.63, 0.73)	<0.001	0.70	(0.63, 0.78)	<0.001
*35–44*.*9*	0.50	(0.47, 0.52)	<0.001	0.57	(0.51, 0.63)	<0.001
*45+*	0.47	(0.44, 0.50)	<0.001	0.56	(0.50, 0.63)	<0.001
**WHO stage at enrollment (w/in 3 mos)**						
*1*	1			1		
*2*	1.01	(0.94, 1.08)	0.844	0.99	(0.92, 1.07)	0.836
*3*	1.14	(1.08, 1.21)	<0.001	1.16	(1.08, 1.24)	<0.001
*4*	1.61	(1.46, 1.78)	<0.001	1.64	(1.53, 1.76)	<0.001
Calendar year*						
*2000–06*	1			1		
*2007–09*	0.34	(0.31, 0.36)	<0.001	1.07	(1.02, 1.12)	0.009
**Highest education level achieved**						
*None*	1			1		
*Some primary*	0.82	(0.71, 0.94)	0.004	0.84	(0.76, 0.93)	0.001
*Some secondary*	0.70	(0.62, 0.80)	<0.001	0.75	(0.67, 0.85)	<0.001
*Some College/University or technical*	0.67	(0.60, 0.76)	<0.001	0.68	(0.59, 0.79)	<0.001
*Missing or N/A*	0.79	(0.68, 0.91)	0.001	0.780	(0.70, 0.87)	<0.001

*At enrollment or ART initiation

** Results from a multivariable semiparametric proportional hazards model with a gamma-distributed shared frailty according to EA-IeDEA program.

While this analysis focused primarily on facility-level factors, various patient-level factors were also significant, and include younger age, more advanced HIV disease based on WHO stage, and lower educational level were all associated with increased hazards of LTFU. LTFU in the pre-ART follow-up period was more frequent among men and patients enrolled prior to 2007. Conversely, LTFU was slightly higher in the post-ART period among those initiating ART more recently (2007–2009 vs. 2000–2006). Tertiary care, the ability to identify patients who transferred in (electronic database), and having a waiting list for initiating ART were not significantly associated with LTFU in the pre-ART period. The provision of nutritional supplements, having a waiting list for initiating ART, referring TB symptomatic patients to a TB clinic, phone first for outreach, and being male were not significantly associated with LTFU from ART.

## Discussion and Conclusions

In this comprehensive analysis of facility-level factors associated with LTFU in 77,362 patients from 29 facilities across three east African countries, we found numerous facility-level factors that were associated with reduced LTFU: facility open more than 4 mornings per week; provision of care at secondary-level facilities such as district hospitals compared to primary care facilities; provision of nutritional supplements, provision of onsite laboratory services and faster CD4 results; and provision of TB care separate from HIV and ART care. These findings suggest some specific modifiable facility-level strategies that programs and facilities should consider when planning and implementing their services to promote retention in HIV care and in turn, maintenance of viral suppression. We found that LTFU rates were also higher in the pre-ART period compared to the ART period, although this is likely to reduce with progressive adoption and implementation of a “treat all” strategy as recommended by WHO, minimising time between diagnosis and ART initiation [23].

LTFU rates were lower in secondary-level facilities compared to primary care facilities. Other studies have shown that decentralization is associated with improved retention [12, 24], as care that is located closer to the home may result in improved geographical access and decreased transport costs. However, the level of care (tertiary, secondary or primary care) is also be a proxy for other factors including staffing, available resources and services provided. A recent study on patient attitudes towards decentralization suggests that many patients experience less stigma in centralized sites where there is less proximity to their local community and there are also a greater availability of services [25]. We found that provision of additional services, including nutritional support (in pre-ART care only) and laboratory capacity (both pre- and post-ART care) impacted LTFU. While not assessed directly in our study, our findings of higher rates of LTFU among ART patients in tertiary care may be partially be explained by the tendency to refer sicker patients to larger more specialized tertiary facilities which are usually located in larger urban centers and farther from patients’ homes [26]. However, while some individuals may prefer services closer to their homes to minimize travelling, others may purposely avoid clinics close to their homes in order to conceal their status from community members [27].

Facilities that were open more than 4 mornings per week reported fewer losses to follow-up. More available and flexible opening hours [24, 27] may help particularly patients in full time employment keep their clinic appointments, although health care worker shortages may make this difficult to implement. This can be facilitated by task-shifting from clinicians to lower cadres of health workers to increase access [28] together with other strategies such as convenient clinic location and ease of access. The promotion of differentiated care and follow up [29] with less frequent visits for individuals stable and virally suppressed on ART represents a more efficient and cost-effective service provision strategy [30], which minimises the onus on those who have challenges attending the facility during current operating hours.

Provision of onsite laboratory services and faster results turnaround time was associated with reduced LTFU. Our finding largely relates to delays in CD4 testing rather than viral load (as few sites were undertaking routine viral load during the study period), and also may serve as a proxy for facility function and responsiveness. It is recognized that limited human resources, lack of timely CD4 testing (due to broken CD4 machines or missing reagents) [31] and long delays in receiving results can decrease prompt assessment of ART eligibility and initiation [31] and negatively impact retention [15]. With the progressive shift towards implementation of a “treat all” strategy that does not require a CD4 cell count for ART initiation [23], the impact of laboratory access is likely to lessen. Point of care CD4 testing has also been used in many settings to reduce delays in receiving test results and ART initiation [31] as well as improve linkage into care [27]. As viral load monitoring replaces CD4 cell count monitoring as the main approach to assessment of ART response and diagnosis of treatment failure [32], there will need to be an emphasis on reducing turnaround time and improving timely communication of results to patients in order to improve overall retention along the HIV cascade.

Finally, our study found that management of TB within the HIV and ART program rather than in a separate TB programme was associated with a higher hazard of LTFU. HIV-TB co-infection remains a major problem in sub-Saharan Africa and continues to present numerous challenges to HIV treatment programs, particularly in infection control and risks of TB transmission and acquisition in an HIV infected population [25]. In response, there has been a push towards integrated care for HIV and TB [33] (e.g., providing services at single facility) which is associated with increased uptake of HIV testing and TB case detection and also minimises inconvenience of traveling to more than one facility and transport costs [34]. However, integrating care presents several challenges: it may overburden already constrained HIV programs, particularly given the more frequent clinic visits required during TB treatment [35]; increase patient wait times, and present risks in infection control and nosocomial spread of TB to HIV positive patients [34]. In addition, the number of staff trained in HIV-TB co-management may be few and cross-training and the merging of operations may be costly [35]. A recent Ghanaian study noted that while HIV testing increased among TB patients, ART use did not [36] and more losses occurred in an integrated care program versus single service clinics in a South African study [37]. Further studies are needed to explore all aspects of the impact of integrated care on retention [7, 24].

There were several limitations to the present study. The facility-level data available were based on a site assessment that was conducted in late 2009 while the patient-level data was collected from 2000 through early 2009. However, we consider that the critical patient and service delivery factors still apply, and an updated assessment is planned to identify the impact of more recent changes in facility-level factors on retention. There were also several potentially important factors reported in other studies that we did not capture—these include the impact of patient-provider relationships, clinic visit wait times, and transport costs to the clinic [5, 13–15, 24], as well as significant missing data and lack of pre-ART data for one of the Uganda sites. We were not able to exclude impact of potential bias by indication on our findings. For example, it is also possible that our finding of a higher loss to follow up in primary care outreach programs, may have been because the outreach program itself was established as a result of high rates of LTFU. While adjustment for time-dependent factors can help to minimize bias [38], time-updated variables were not available in the present study. Finally, individuals not tracked through outreach may have died and so we may have overestimated LTFU. There is a clear need to distinguish patients who have died from those LTFU for other reasons in order to more accurately estimate retention. This was a large analysis of more than 70,000 patients from 29 sites across three countries in east Africa, and although not based on comprehensive national data, our study population was broadly representative of HIV infected patients’ population of east Africa, with the exclusion of pregnant women and children under the age of 14 years.

Overall our findings support the implementation of several key strategies to improve retention in care: onsite and timely laboratory testing; flexible facility hours and daily clinics, provision of care in secondary level health facilities that can provide a more comprehensive range of services. This will become more critical with continued scale up of ART to all 34 million persons living with HIV. This is particularly the case in light of the increasing burden of co-morbidities, such as tuberculosis, diabetes, or hypertension, and given the move towards more comprehensive reproductive health care to prevent maternal-child HIV transmission. If we are to meet the UNAIDS 2020 targets and fully realize the individual and public health benefits of ART, health care facilities must be both evidence-based and patient –centered, along each stage of the care-prevention continuum.

## Supporting Information

S1 File Model for the cause-specific hazard of LTFU pre- and post-ART. (DOC)
