# Supplementary material for: Facility-Level Factors Influencing Retention of Patients in HIV Care in East Africa
Source: PLoS One. 2016 Aug 10;11(8):e0159994. doi: 10.1371/journal.pone.0159994 (PMC4980048; doi:10.1371/journal.pone.0159994)
Supplement: S1 File — (DOC) [file pone.0159994.s001.doc]

**Appendix A: Model for the cause-specific hazard of LTFU pre- and post-ART**


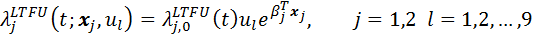


Where
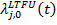
 is the baseline cause-specific hazard for the *j*-th analysis period (*j*=1 for the pre-ART and *j*=2 for the post-ART period), *u*_l_ is the shared-frailty, i.e. the random effect of the specific EA-IeDEA program (*n*=9), which is assumed to follow a gamma distribution with mean 1 and variance *θ* to be estimated from the data and
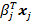
 is the sum of the effect*predictor value products for each analysis period:


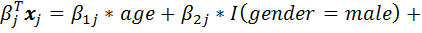
…

Both individual level (age, gender, WHO stage, highest educational level achieved, calendar year of enrollment or ART initiation) and facility level (level of care, waiting lists, HIV-RNA and CD4 exam availability, operating schedules etc.) predictors were considered in the multivariable analysis.
